# Rare case report: Liver metastasis from cervical adenocarcinoma presenting with cystic mass and obstructive jaundice

**DOI:** 10.3389/fonc.2025.1558946

**Published:** 2025-05-30

**Authors:** Jin Li, Xin Wei, Le Luo

**Affiliations:** ^1^ Department of Ultrasound, Deyang People’s Hospital, Deyang, Sichuan, China; ^2^ Medical Records Statistics Management Section, Deyang People’s Hospital, Deyang, Sichuan, China

**Keywords:** liver metastases, hepatic cystic neoplasms, cervical cancer, contrast-enhanced ultrasound, case report

## Abstract

Liver metastases can originate from primary tumors in various organs; however, metastasis from cervical cancer to the liver is rare. Cervical cancer patients with distant metastases have a poor prognosis and reduced survival rates. This report describes a case of a cystic liver mass with obstructive jaundice, observed four years after resection of cervical adenocarcinoma. The lesion lacked typical imaging characteristics of hepatic metastases and was initially suspected to be a biliary neoplasm. A contrast-enhanced, ultrasound-guided needle biopsy was performed to confirm the diagnosis. Histopathological analysis confirmed adenocarcinoma of the liver, and immunohistochemical staining suggested a uterine or adnexal origin. Considering the patient’s surgical history, the final diagnosis was liver metastasis originating from cervical adenocarcinoma. This report reviews relevant literature to discuss the clinical features, diagnostic challenges, and therapeutic strategies for liver metastasis of cervical cancer.

## Introduction

Metastatic liver tumors can originate from malignancies in nearly any organ system. However, liver metastasis from cervical cancer is extremely rare, with an incidence of approximately 2.32% among cervical cancer patients (1, 2). Distant metastasis in cervical cancer is associated with poor prognosis, with a five-year survival rate of 17.6% compared to 91.9% in patients with localized disease (1, 3, 4). According to the Surveillance, Epidemiology, and End Results (SEER) database data, approximately 30% of patients with cervical cancer develop distant metastases. The most frequent metastatic sites among these are the lungs (65%), bone (35.9%), liver (32.2%), and brain (5.6%) (1, 2, 5). Distant metastasis of cervical cancer is usually associated with poor prognosis and reduced survival, especially in patients with multi-organ metastasis. This report describes a rare case of liver metastasis from cervical adenocarcinoma, manifesting as a cystic liver mass accompanied by biliary obstruction. A detailed evaluation of the clinical presentation, diagnostic workup, and therapeutic strategies is presented, along with a review of relevant literature.

## Case presentation

**Figure 1:**
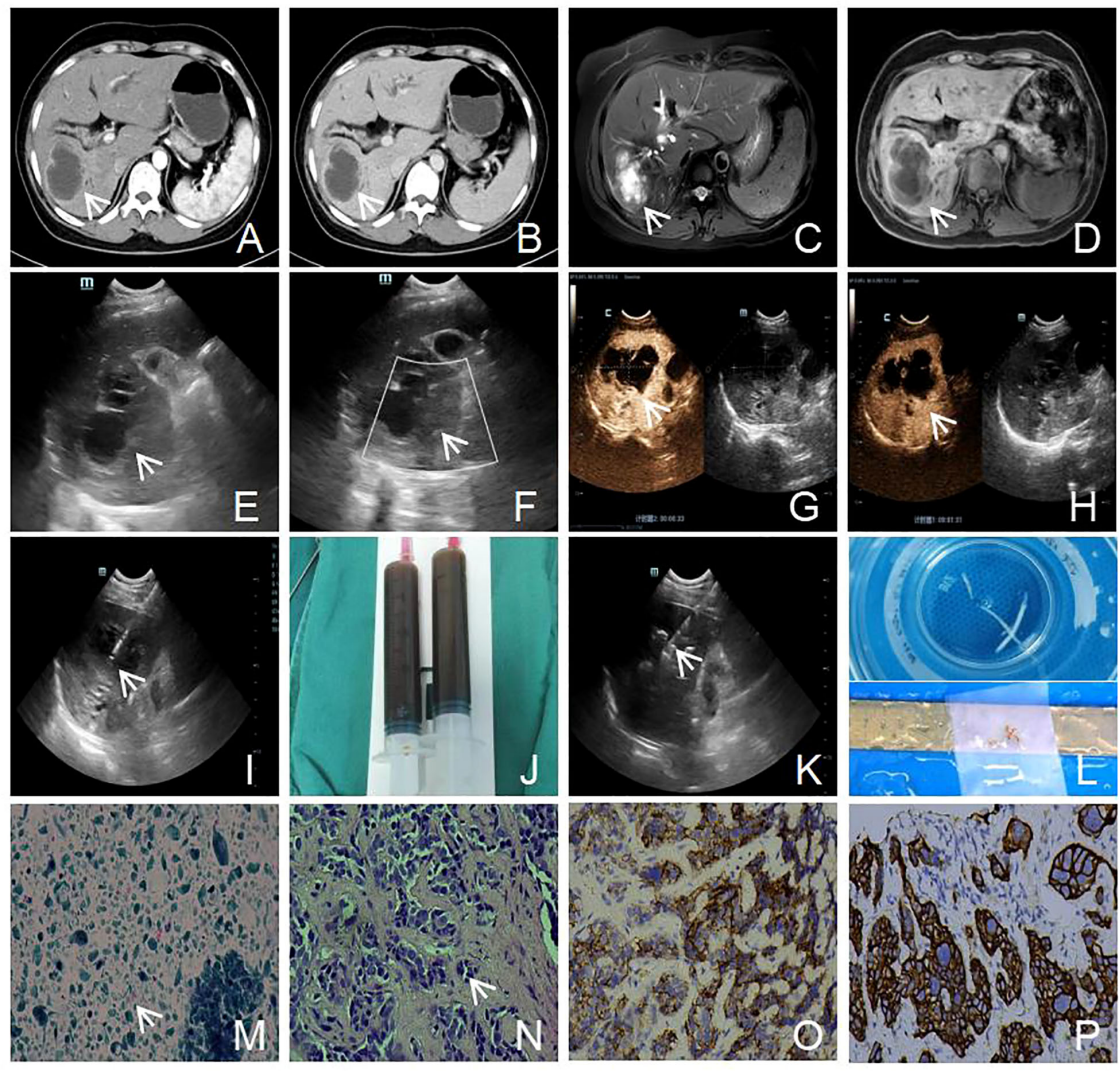
Imaging and histopathological features of a hepatic mass in a 48-year-old patient. **(A, B)** Contrast-enhanced CT scans demonstrate a hypodense lesion in the right hepatic lobe with peripheral ring enhancement and non-enhancing core, associated with intrahepatic bile duct dilation (white arrow). **(C, D)** Contrast-enhanced MRI shows a thick-walled cystic lesion in the right hepatic lobe with peripheral ring enhancement (white arrow). **(E, F)** Ultrasound examination shows a cystic-solid mass with ill-defined margins; color Doppler imaging demonstrates no detectable intralesional blood flow (white arrow). **(G, H)** Contrast-enhanced ultrasound reveals rapid thin peripheral hyperenhancement without washout and absence of internal enhancement (white arrow). **(I, J)** Ultrasound-guided cyst aspiration using a 17G coaxial needle extracted 40 mL of dark-green viscous fluid (white arrow). **(K, L)** Subsequent core needle biopsies from the mass periphery and central region were obtained using an 18G semi-automatic device to collect three tissue cores (white arrow). **(M)** Cytological examination of the cystic fluid shows a few atypical keratinized cells in the cystic background (H&E staining, 200x), (white arrow). **(N)** Histological section shows tumor cells arranged in nests with marked nuclear pleomorphism, irregular contours, and hyperchromasia (H&E staining, 200x), (white arrow). **(O)** Immunohistochemical staining for CA125 shows positive membranous expression in cancer cells (EnVision staining, 200x). **(P)** Immunohistochemical staining for CK7 shows positive cytoplasmic expression in cancer cells (EnVision staining, 200x).

**Figure 2:**
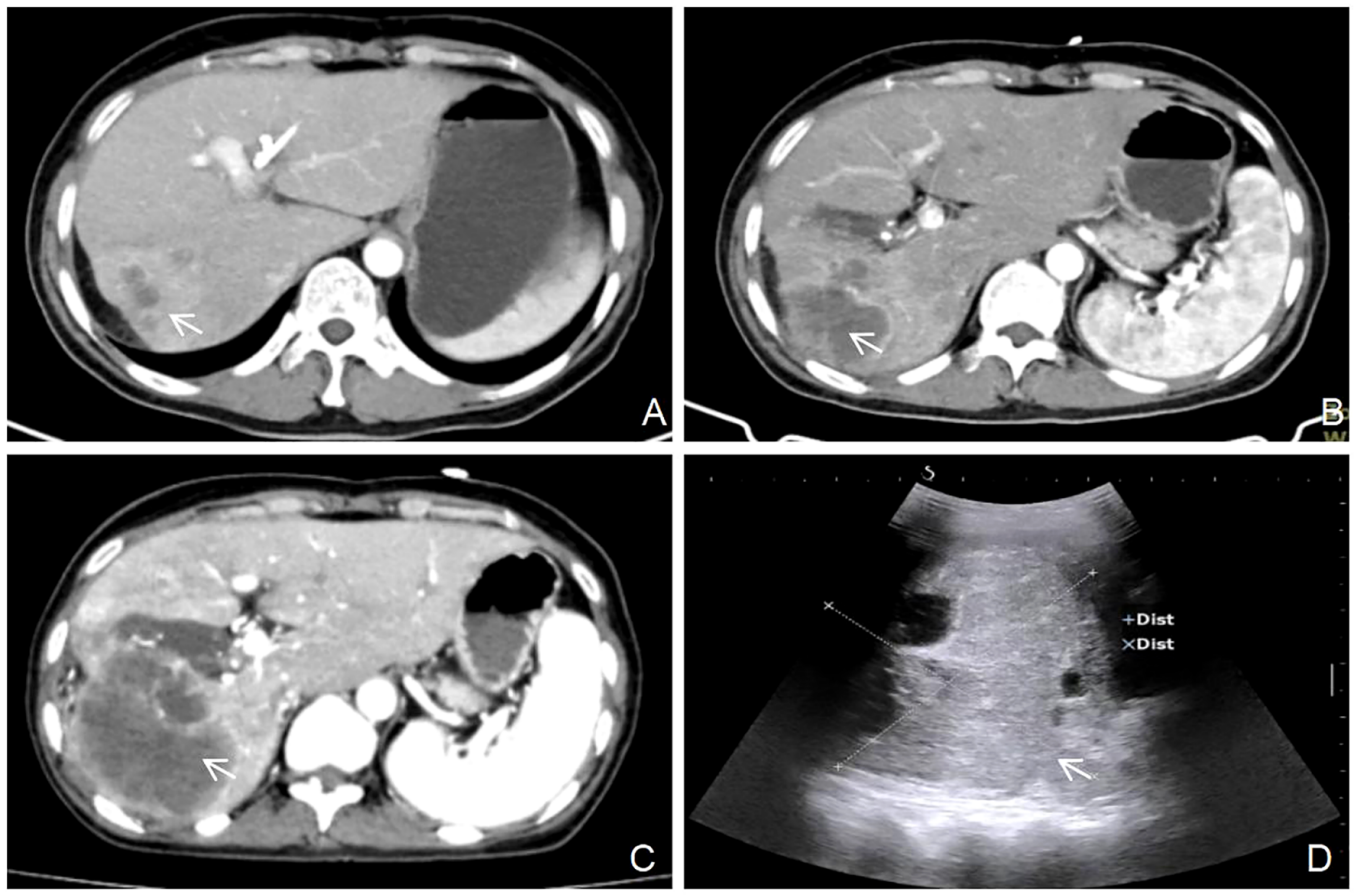
Serial imaging evaluation of tumor progression following chemotherapy completion. **(A)** after 4 cycles of chemotherapy, size of the tumor: 52 mm x 48 mm (white arrow). **(B)** 3 months post-chemotherapy, size of the tumor: 67mm x 64 mm (white arrow). **(C)** 6 months post-chemotherapy, size of the tumor: 86 mm x 84 mm (white arrow). **(D)** 9 months post-chemotherapy, size of the tumor: 109 mm x 106 mm (white arrow).

A 48-year-old woman with a history of cervical adenocarcinoma resected four years before was admitted with complaints of abdominal distension and jaundice persisting for over one month. Computed tomography (CT) (**Figure 1A, B**) revealed a low-density mass in the right hepatic lobe, measuring approximately 53 mm × 40 mm, with annular enhancement, hilar bile duct obstruction, and significant intrahepatic bile duct dilatation. After exclusion of surgical contraindications, the patient underwent percutaneous transhepatic cholangiographic drainage (PTCD). One month after PTCD, the patient developed intermittent abdominal pain lasting over 10 days, accompanied by radiating pain in the lower back. There were no clear aggravating or relieving factors, and mild scleral and skin jaundice persisted. Laboratory testing showed elevated tumor markers: carcinoembryonic antigen (CEA) 25.98 ng/mL, carbohydrate antigen CA125 562.70 U/mL, carbohydrate antigen CA15-3 38.90 U/mL, carbohydrate antigen CA19-9 69.83 U/mL, squamous cell carcinoma antigen (SCC) 34.06 ng/mL, cytokeratin 19 fragments (CYFRA21-1) 44.19 ng/mL, and PIVKA-II 919 mAU/mL. Alpha-fetoprotein (AFP) remained within normal limits at 1.80 ng/mL. Liver function tests showed elevated total bilirubin (TBIL; 29.0 µmol/L), direct bilirubin (DBIL; 21.4 µmol/L), alanine aminotransferase (ALT; 94 U/L), aspartate aminotransferase (AST; 68 U/L), and γ-glutamyl transpeptidase (GGT; 465 U/L). A complete blood count revealed a white blood cell count (WBC) of 19.08 × 10^9^/L, neutrophil percentage (NEUT%) of 88.7%, and neutrophil absolute count (NEUT#) of 16.93 × 10^9^/L. Magnetic resonance imaging (MRI; **Figure 1C, D**) demonstrated multiple hepatic lesions, the largest located in the right lobe and hepatic hilum, measuring approximately 68 mm × 69 mm × 64 mm. The lesion showed annular enhancement, portal vein branch truncation, and intrahepatic bile duct dilation. Grayscale ultrasound (**Figure 1E, F**) revealed multiple hepatic masses, the largest measuring 81 mm × 69 mm in the right lobe and hepatic hilum. Contrast-enhanced ultrasound (**Figure 1G, H**) revealed rapid peripheral enhancement in the arterial phase with slow fading to isoenhancement during the parenchymal and delayed phases. A central non-enhancing region (70 mm × 50 mm) without contrast perfusion was identified. To confirm the diagnosis and formulate the treatment plan, an ultrasound-guided liver biopsy was performed (**Figure 1I, J**). Approximately 40 mL of cystic fluid was aspirated (**Figure 1K**), and fish-flesh-like tissue was obtained from the tumor edge (**Figure 1L**). Histopathology (**Figure 1M, N**) confirmed adenocarcinoma. Immunohistochemistry (**Figure 1O, P**) showed positivity for CK7, CK19, CA125, GS, PAX8, and p16; Ki-67 proliferation index was 25%. Negative markers included CK20, Villin, CDX2, Hepa, and WT1 (Wilm tumor). These findings were consistent with moderately differentiated adenocarcinoma of Müllerian origin. In light of the patient’s history, a diagnosis of hepatic metastasis from cervical adenocarcinoma was established. After ruling out chemotherapy contraindications, the patient received GP regimen chemotherapy: gemcitabine (1.5 g on days 1 and 8) plus cisplatin (30 mg on days 1–3). After one cycle of treatment, clinical symptoms, including abdominal pain and jaundice, improved. After four cycles, symptoms improved significantly, and follow-up CT (**Figure 2A**) showed tumor shrinkage. However, follow-up imaging three months post-chemotherapy (**Figure 2B**) revealed increased tumor volume. Six months post-chemotherapy (**Figure 2C**), the tumor volume increased further, and by nine months post-chemotherapy (**Figure 2D**), the mass measured approximately 109 mm × 106 mm. The patient’s condition continued to deteriorate, resulting in death from multiple organ failure one month later.

## Discussion

Cervical cancer is one of the most common malignant tumors affecting the female reproductive system. Although its global incidence and mortality have decreased in recent years due to widespread human papillomavirus (HPV) vaccination and routine cervical cancer screening, patients with advanced disease still face a high risk of metastasis. This is particularly true in developing countries, where both incidence and mortality rates remain elevated (1). According to GLOBOCAN 2022, cervical cancer was the fourth most commonly diagnosed and the third leading cause of cancer-related death among women worldwide, with approximately 660,000 new cases and 350,000 deaths reported in 2022 (6).

To date, most studies on liver metastasis from cervical cancer are based on data from the SEER database. These studies have focused on patterns of distant metastasis, prognostic factors, and treatment outcomes, providing important information for clinical decision-making and survival prediction (1, 2, 5). These findings emphasize the importance of early detection and comprehensive management. However, case reports specifically addressing hepatic metastasis from cervical cancer remain limited. To further investigate the rarity of this condition, a PubMed search was conducted for reports published between 1950 and 2025 using the terms “hepatic metastases” and “cervical cancer”. After careful screening, only four relevant case reports were identified (7–10). Ultimately, five cases, including the present one, were analyzed (**Table 1**). The mean patient age was 50 years, with a median of 49 years. Clinical presentations included obstructive jaundice (1 case), liver pseudocyst (1 case), hepatogastric fistula (1 case), portal vein tumor embolism (1 case), and cystic liver masses with obstructive jaundice (1 case). All cases were evaluated by multiple imaging modalities and were confirmed by pathological examination: one *via* surgical resection, one *via* endoscopic biopsy, and three *via* percutaneous needle biopsy. Of the five reported cases, all involved cervical squamous cell carcinoma metastasizing to the liver. On the other hand, this case involved cervical adenocarcinoma with liver metastasis, a less frequently reported histological subtype. All patients in the reviewed cases received palliative chemotherapy, which controlled the disease to a certain extent but did not significantly improve long-term outcomes. The overall prognosis remained poor, with significant impairment in quality of life.

**Table 1:** Summary of 6 cases of liver metastasis of cervical cancer in contemporary literature.

Author/year of publication	Age/years	Clinical picture	Clinical signs and symptoms	Imaging Studies	Methods of Diagnosis	Pathological diagnosis	Treatment	Outcomes
Raggio et al., (7)	38	Obstructive jaundice	Jaundice; nausea; vomiting; abdominal pain	US: Obstructive jaundice	Chiba needle biopsy	Squamous cell carcinoma	Radiotherapy and Chemotherapy	The condition improved, but the final outcome was not reported
Alsolaiman et al., (8)	58	Hepatic pseudocyst	Abdominal pain; fullness following eating; worsening heartburn; increasing abdominal	CT:Diffuse polycystic disease of the liver	Ultrasound-guided the cyst was punctured and drained	Squamous cell carcinoma	Unreported	Unreported
Narayanan et al., (9)	58	Hepatogastric fistula	Abdominal pain; distension	PET-CT:Heterogeneous enhancement lesions in the liver and lesser curvature of the stomach	An upper gastrointestinal endoscopy	Squamous cell carcinoma	Chemotherapy	Condition deteriorated and death
Nakamura et al., (10)	49	Portal vein tumor thrombosis	Unreported	CT:Two irregular tumors in the liver’s segments 2 and 6	Surgical resection	Squamous cell carcinoma	Liver resection and Chemotherapy	He died 10 months later
Our case (2024)	48	Obstructive jaundice; Hepatic pseudocyst	Jaundice;nausea; vomiting; abdominal pain;	MRI: abnormal signal mass shadow in the right lobe of liver and hepatic hilum; US: cystic solid mass in the right lobe of the liver	Ultrasound-guided needle biopsy	Adenocarcinoma	Chemotherapy	He died 11 months later

The clinical manifestations of liver metastasis from cervical cancer vary. Some patients, particularly in the early stages, may remain asymptomatic. On the other hand, patients with extensive or extrahepatic disease may experience non-specific symptoms such as lower back pain, nausea, vomiting, anorexia, and abdominal distension. Obstructive jaundice can occur when hepatic lesions involve the biliary tract (7). Tumor markers such as CEA, CA125, and CA19–9 may be elevated, but their sensitivity is limited, and not all patients exhibit abnormalities (10). Therefore, the diagnosis of liver metastasis from cervical cancer primarily relies on imaging and histopathological confirmation. Commonly used imaging techniques include ultrasonography, CT, MRI, and PET-CT (1–3, 10). Ultrasound serves as a first-line screening tool capable of detecting cystic or solid hepatic lesions. CT and MRI offer more detailed anatomical resolution, with MRI providing particularly high sensitivity and specificity for distinguishing between benign and malignant lesions. PET-CT is valuable for systemic metastasis evaluation, especially in patients with suspected multi-organ involvement. Among diagnostic approaches, ultrasound-guided needle biopsy is considered the gold standard for confirming hepatic metastasis from cervical cancer due to its safety, minimally invasive nature, and high diagnostic accuracy (4, 8).

Patients with metastatic cervical cancer are generally considered incurable; however, treatment can help alleviate symptoms and prolong survival. Therapeutic strategies for hepatic metastases from cervical cancer include systemic therapy and local interventions. Treatment selection depends on the patient’s overall health, the extent of cancer spread, and the number and location of hepatic lesions (1). For patients with multiple extrahepatic metastases, systemic chemotherapy remains the mainstay of treatment. According to the gynecologic oncology group (GOG) 240 trial, the standard regimen for metastatic, persistent, or recurrent cervical cancer includes platinum-based chemotherapy combined with the angiogenesis inhibitor bevacizumab (2, 11, 12). Kim et al. demonstrated that chemotherapy significantly improves median survival in patients with liver metastases compared to those who do not receive treatment (1, 13). Similarly, Tewari et al. found that adding bevacizumab to chemotherapy significantly improves overall survival compared to chemotherapy alone (11). Local treatments, such as partial hepatectomy, stereotactic body radiation therapy (SBRT), and interventional procedures, can be considered in selected patients. In selected patients with isolated metastases confined to a single hepatic lobe, partial hepatectomy may provide a survival benefit (14, 15). However, the majority of patients present with multifocal hepatic and extrahepatic metastases, making them ineligible for surgical resection. SBRT has demonstrated efficacy in achieving local control in patients with oligometastatic cervical cancer and may contribute to prolonged survival (16, 17). Minimally invasive and repeatable interventional techniques, such as microwave ablation and transarterial chemoembolization (TACE), are increasingly employed to treat primary and secondary liver tumors in patients ineligible for surgery (16, 18). Moreover, Yttrium-90 radioembolization (Y-90 RE), in combination with immunotherapy, has shown efficacy in controlling hepatic metastases in patients who experienced progression following chemotherapy and TACE, offering a potential option for aggressive metastatic disease (19). In this case, the patient presented with multiple hepatic metastases and was not a candidate for a local treatment approach. Systemic chemotherapy was administered, yielding initial symptomatic and radiologic improvement; however, the disease then progressed, highlighting the aggressive nature and poor prognosis of liver metastatic cervical cancer. These cases highlight the need for individualized, multimodal treatment strategies to improve response and prolong survival.

## Conclusions

Although liver metastasis from cervical cancer is relatively rare, it is associated with a poor prognosis and significantly reduced survival. Imaging modalities—such as ultrasound, CT, MRI, and PET-CT—play a key role in diagnosis, while ultrasound-guided needle biopsy remains the gold standard for definitive pathological confirmation. Treatment strategies should be tailored to the patient’s clinical status, tumor burden, and extent of metastasis. Combining systemic chemotherapy and local therapies, such as SBRT or TACE, may offer clinical benefit. However, patients with multiple extrahepatic metastases continue to have limited therapeutic options and poor survival outcomes. Further studies are needed to elucidate the molecular mechanisms of cervical cancer liver metastasis and to develop more effective, individualized treatment approaches.

## Data Availability

The datasets presented in this study can be found in online repositories. The names of the repository/repositories and accession number(s) can be found in the article/Supplementary Material.
